# Type 1 neovascularization with polypoidal lesions complicating dome shaped macula

**DOI:** 10.1186/s40942-015-0008-5

**Published:** 2015-07-09

**Authors:** Jonathan Naysan, Kunal K Dansingani, Chandrakumar Balaratnasingam, K Bailey Freund

**Affiliations:** 1 Vitreous Retina Macula Consultants of New York, 460 Park Avenue, Fifth Floor, New York, NY 10022 USA; 2 The LuEsther T. Mertz Retinal Research Center, Manhattan Eye, Ear and Throat Hospital, New York, NY USA; 3 Department of Ophthalmology, New York University School of Medicine, New York, NY USA; 4 Department of Ophthalmology, North Shore-Long Island Jewish Health System, New York, NY USA; 5 Moorfields Eye Hospital, London, UK; 6 Department of Physiology and Pharmacology, Lions Eye Institute, University of Western Australia, Crawley, Australia

**Keywords:** Dome-shaped macula, Polypoidal, Polypoidal choroidal vasculopathy, Polypoidal choroidal neovascularization, High myopia, Neovascularization, Imaging

## Abstract

Dome-shaped macula is described as an inward bulge of the macula within a posterior staphyloma in highly myopic eyes. Choroidal neovascularization is a known complication that can cause visual loss in dome-shaped macula. Herein, we describe a patient who presented with features of polypoidal choroidal neovascularization that developed on a background of high myopia with dome-shaped macula.

## Background

Dome-shaped macula (DSM) is an inward bulge of the macula within a posterior staphyloma in highly myopic patients [1]. Recently, Imamura et al. [2] utilized enhanced depth optical coherence tomography (EDI-OCT) to demonstrate that the anterior bulge was consequent to relative increases in scleral thickness beneath the macula. Gaucher et al. [1] reported that the prevalence of dome-shaped macula was approximately 10.7% in highly myopic eyes. However, recent reports have utilized multimodal imaging to show that the rate of DSM is higher and in the range between 15 and 20% [3, 4].

Choroidal neovascularization (CNV) is a known complication of DSM and occurs at a rate of approximately 20% [4, 5]. Deobhakta et al. [6] recently demonstrated that some eyes with DSM will manifest a shallow pigment epithelial detachment (PED) overlying an area of abrupt change in choroidal thickness typically occurring at the edge of the dome. They hypothesized that some of these flat irregular PEDs might harbor type 1 neovascularization. Herein, we describe the multimodal imaging findings in a patient with DSM who presented with type 1 neovascularization with polypidal lesions. To our knowledge, polypoidal type 1 neovascularization has not been documented in the setting of DSM.

## Case presentation

A 50-year-old white female with a history of high myopia presented with metamorphopsia and visual deterioration in her left eye that had developed gradually over many months. She had previously been diagnosed with central serous chorioretinopathy and had received photodynamic therapy to the left eye after which two intravitreal injections of bevacizumab were administered (last treatment was over 1 year previously). She was taking metformin for glucose intolerance and was otherwise healthy.

Visual acuities were ^20^/_250_ in the right eye, improving to ^20^/_100_ with pinhole and ^20^/_800_ in the left eye, improving to ^20^/_100_ with pinhole. Her refractive error was −7.5 and −9.0 diopters right and left, respectively.

**Figure 1:**
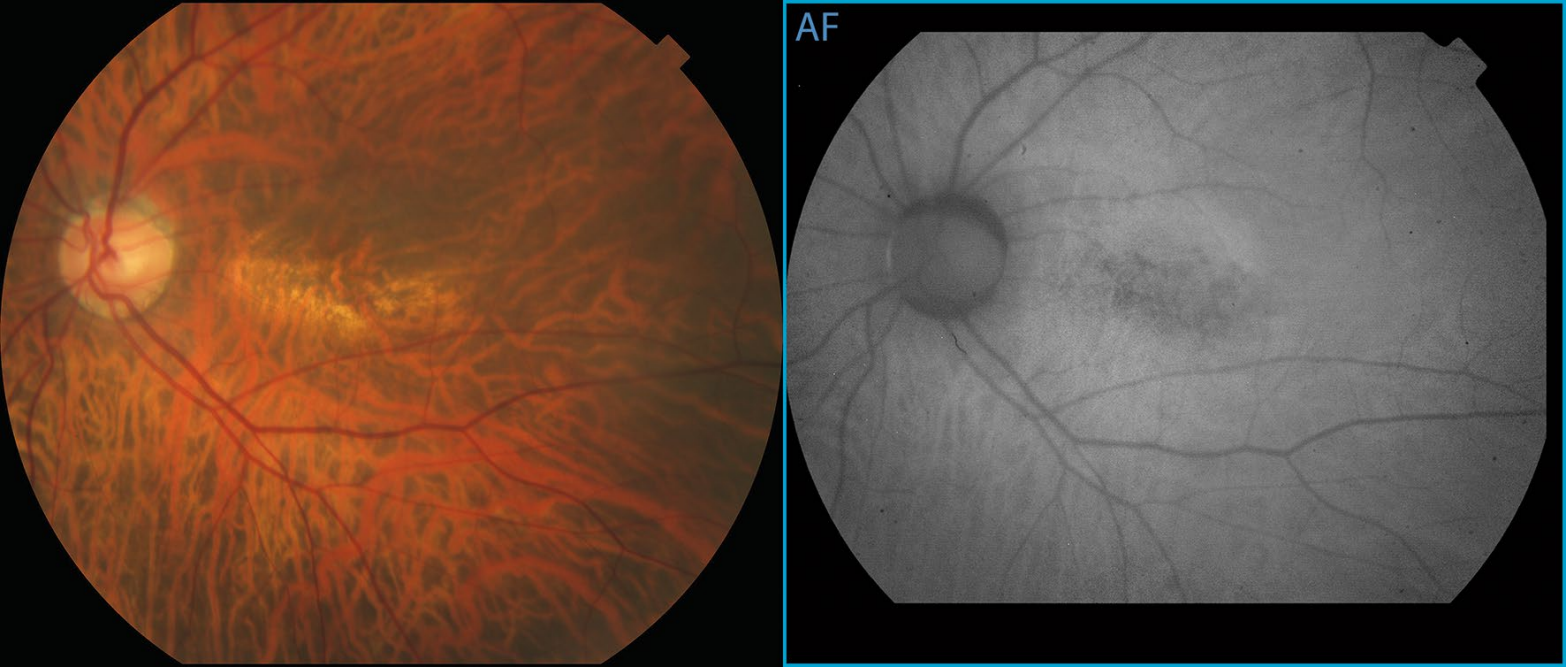
Color photograph and fundus autofluorescence images of the posterior pole of the* left* eye show a rectangular area of mottled retinal pigment epithelial atrophy arranged with its long axis aligned horizontally.

The anterior segments were unremarkable and intraocular pressures were 15 mmHg in both eyes. Ophthalmoscopic examination showed peripapillary atrophy and retinal pigment epithelial changes that were consistent with myopic degeneration (Figure 1). There was no hemorrhage.

**Figure 2:**
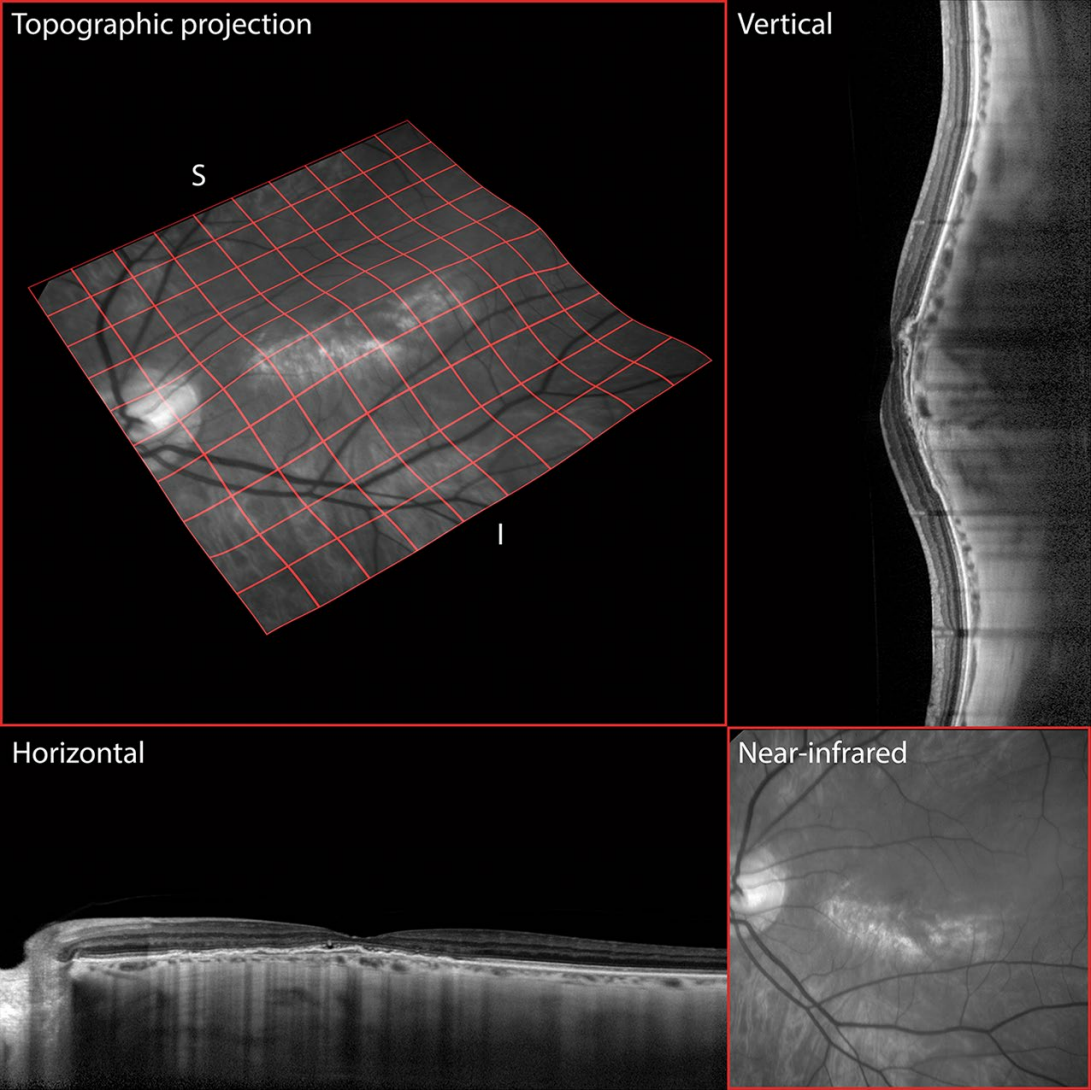
Topological representations of the posterior pole. Vertical and horizontal raster spectral domain optical coherence tomography line scans show that the long axis of the elliptical dome lies in the horizontal meridian, as demonstrated also by a quad-mesh projection onto an elevation-mapped near-infrared image plane. The region of atrophy lies at the inferior border of the crest of the elliptical dome, where the choroid is thinnest.

**Figure 3:**
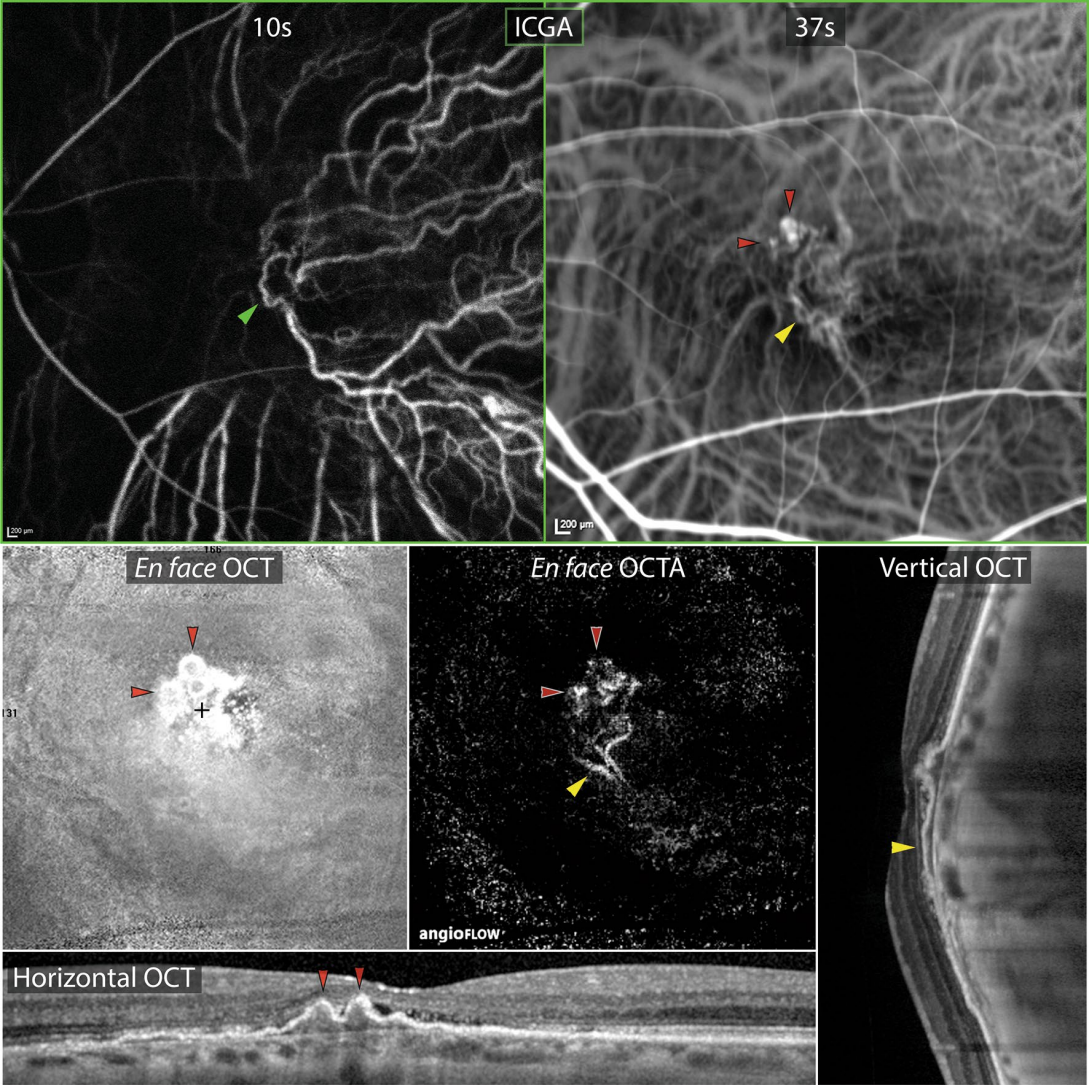
Multimodal imaging findings of choroidal neovascularization. Early and transit phase indocyanine green angiographic frames demonstrate perfusion of a choroidal vascular loop at the fovea (*green arrowhead*) followed by hyperfluorescence of at least three polypoidal lesions (*red arrowheads*) and their feeding network (*yellow arrowhead*). Magnified horizontal and vertical raster enhanced depth spectral domain optical coherence tomographic (OCT) line scans through these areas show the polypoidal lesions as peaked pigment epithelial detachments (PEDs) and the feeding network as an adjacent shallow irregular PED. The presence of pathologically dilated choroidal vessels is noted with overlying loss of choriocapillaris tissue, especially at the crest of the dome. *En face* OCT (3 × 3 mm) through the shallow PED reveals the spherical morphology of at least three polypoidal lesions. *En face* OCT angiography (3 × 3 mm) through the PED isolates the type 1 neovascular tissue from the rest of the choroid and shows significant flow through the feeder vessels and within the polypoidal lesions.

Horizontal OCT (Heidelberg Spectralis, Heidelberg Engineering, Germany) raster scans did not overtly illustrate any dome configuration of the patient’s macula. However, a combination of horizontal and vertical raster scans enabled a radially asymmetric three-dimensional dome-shaped configuration to be appreciated (Figure 2). Spectral domain OCT showed thin choroids in both eyes with subfoveal choroidal thickness of 98 and 95 um, respectively. The vertically oriented OCT line scans showed an abrupt change in choroidal thickness occurring at the inferior edge of the dome (Figure 3). EDI-OCT confirmed an increase in scleral thickness beneath the site of the macular dome.

A complex irregular pigment epithelial detachment was noted at the left macula consistent with a polypoidal lesion and its associated type 1 branching vascular network (BVN) (Figures 2, 3). Shallow subretinal fluid was also seen on these scans. Indocyanine green angiography (TRC-50FX, Topcon Corporation, Tokyo, Japan) and split spectrum amplitude decorrelation angiography (Avanti, Optovue, Fremont, CA, USA) demonstrated the polypoidal lesion and its corresponding feeding vessels (Figure 3). The ICGA also showed relatively larger caliber choroidal vessels in the thicker choroid at the superior edge of the dome.

DSM is associated with high myopia [1] and previous reports have shown that this diagnosis can be missed if the macula is evaluated with a single OCT scan orientation. Liang et al. [4] reported that vertical and horizontal OCT scans are necessary to achieve diagnostic sensitivity for DSM. In their paper, they showed that of those eyes with DSM, the diagnosis could be made using only the vertical section in 77% of cases and using only the horizontal section in 2% of cases. These findings are reflected in our patient in whom the diagnosis of DSM was confirmed only after integration of vertical and horizontal OCT scans to construct a three-dimensional structure.

Vision loss from DSM can be consequent to serous neurosensory detachment masquerading as central serous chorioretinopathy [5]. Foveal retinoschisis [4, 7] and choroidal neovascularization [1, 7] are other known complications that can arise during the natural course of DSM. The rate of CNV in DSM is approximately 20%, however, little information is available concerning the morphology of these lesions. Utilizing OCT angiography and other imaging modalities, we show that CNV in DSM can manifest a polypoidal configuration. Polypoidal neovascular changes have also been described in central serous chorioretinopathy [8, 9], choroidal nevi [10, 11], peripheral exudative hemorrhagic chorioretinopathy [12] and optic nerve melanocytoma [13].

## Conclusion

Scleral thickness at the site of DSM is increased in contradiction to what is usually found in high myopia [14]. Previous authors used this observation to postulate that control of ocular expansion in myopia may be more complex than initially proposed [2]. Polypoidal changes to type 1 neovascularization in the setting of DSM may reflect the unique stretch and mechanical forces being applied to the macula and choroid in this condition. It will be important to study other cases of CNV in DSM to validate this hypothesis.

### Consent

Written informed consent was obtained from the patient for publication of this Case report and any accompanying images. A copy of the written consent is available for review by the Editor-in-Chief of this journal.

## Abbreviations

DSM: dome-shaped macula
EDI-OCT: enhanced depth imaging optical coherence tomography
CNV: choroidal neovascularization
PED: pigment epithelial detachment
OCT: optical coherence tomography
BVN: branching vascular network
